# The Control of Stress Induced Type I Diabetes Mellitus in Humans through the Hepatic Synthesis of Insulin by the Stimulation of Nitric Oxide Production

**Published:** 2012-09

**Authors:** Rajeshwary Ghosh, Rabindra Bhattacharya, Gorachand Bhattacharya, Asru K. Sinha

**Affiliations:** 1 *Sinha Institute of Medical Science & Technology, Garia 700084, India;*; 2 *Calcutta National Medical College and Hospital, Kolkata-700 014, India*

**Keywords:** dermcidin isoform 2, hepatic insulin synthesis, nitric oxide, sodium nitroprusside, type IB diabetes mellitus

## Abstract

The role of stress induced development of Type-1 diabetes mellitus (T1DM) as opposed to autoimmunity remains obscure. It has been reported that a stress induced protein, identified to be dermcidin isoform 2 (dermcidin) inhibited insulin synthesis in both the pancreatic β cells and the hepatic cells. As dermcidin effect could be neutralized by the increased production of systemic nitric oxide (NO), investigations were carried out to determine the feasibility of controlling stress induced T1DM through the neutralization of dermcidin by systemic increase of NO. To determine the role of plasma dermcidin level in T1DM subjects (n=45), if any, when the plasma dermcidin level were determined, it was found that the protein level was increased in 65% of the participating volunteers. Efforts were made to normalize the plasma glucose level (median=175 mg/dL) in these T1DM subjects by systemic increase of NO by applying a cotton pad containing 0.28mmol sodium nitroprusside on the abdominal skin. It was found that the systemic increase of NO level decreased the blood glucose level of 275 mg/dL (median) to 115 mg/dL (median) in these volunteers within 24 h with concomitant increase of plasma insulin level from 7.5 μunits/dL to 101 μunits/dL at the same time. The increase of plasma insulin level was accompanied by the decrease of dermcidin level of 124.5 nM to 18 nM with increase of NO from 0.43 ± 0.19 nM to 4.1 ± 1.56 nM. The results suggested that the stress induced T1DM by dermcidin could be controlled by the systemic increase of NO which in consequence led to increased synthesis of insulin.

## INTRODUCTION

Type I diabetes mellitus (T1DM), is an insulin dependent condition which results in hyperglycemia due to the destruction of pancreatic β cells that synthesize and secrete the hypoglycemic protein, an essential hormone for carbohydrate metabolism (1). It is currently believed that the condition develops as several risk factors including immunologic, genetic, and stresses of environmental origin, act synergistically that ultimately result in the development of T1DM (2, 3) due to the destruction of pancreatic β cells making the system completely insulin deficient resulting in hyperglycemia in this condition. In the context of the stress induced development of T1DM, it has been reported that a stress induced protein of Mr.11kDa identified to be dermcidin isoform 2 (dermcidin), is produced both in the pons of the brain (4) and in the endothelial cells under stress induced by acute hypoxia and by tobacco smoke (unpublished). It should also be mentioned here that the smoking of tobacco is reported to be one of the major risk factors of diabetes mellitus (5). It was found that dermcidin inhibited the glucose induced synthesis and secretion of insulin in the pancreatic islets of Langerhans through the inhibition of glucose transport in the islets and hepatic cells (6, 7). Since glucose itself induces the synthesis and secretion of insulin in the pancreatic β cells (8), the dermcidin induced inhibition of glucose transport resulted in the inhibition of both synthesis and secretion of the hormone (6).

Until recently it was believed that the pancreatic cells in the adult mammals were the only cells which were capable of synthesizing and secreting insulin when stimulated by glucose. In contrast, we have recently demonstrated that not only the hepatocytes from the adult mice were capable of synthesizing and secreting insulin in the presence of glucose but the actual amounts of the hormone produced in the liver cells was at least 10 times greater than that produced in the pancreatic cells on weight by weight basis (9). However unlike the pancreatic cells, the liver cells in alloxan induced diabetic mice were not destroyed, in that, they were capable of synthesizing and secreting insulin through the glucose induced expression of two non allelic proinsulin genes even when the ability of the pancreatic cells to synthesize and secrete insulin were completely destroyed (7).

In this context, we have also previously reported that the stimulation of systemic NO synthesis not only reduced the increased plasma dermcidin to normal level, but the oxide was found to restore the synthesis and secretion of insulin through the neutralization of the dermcidin induced inhibition of glucose transport in the islets of Langerhans and in the hepatocytes (6, 7).

In the context of the role of dermcidin in the inhibition of insulin synthesis, we have also reported that the increase of systemic dermcidin level inhibited insulin synthesis not only in the pancreatic cells as described above (6), but also in the hepatocytes in alloxan induced diabetic mice (7). More interestingly it was possible to control hyperglycemia in the alloxan induced diabetic mice through the hepatic insulin synthesis by neutralizing the inhibitory effect of dermcidin on the glucose transport due to the stimulation of the systemic NO synthesis in the absence of external insulin administration (7).

Although the exact mechanism of the development of T1DM remains obscure (10), the destruction of the pancreatic β cells by autoimmunity is believed to be one of the major causes leading to the condition (11, 12). In contrast, we have recently reported that increased systemic level of dermcidin might also contribute to the development of T1DM both in the animal model (7) as well as in humans suffering from coronary artery disease where an acute T1DM like condition is known to develop, albeit temporarily, due to severe impairment of systemic insulin synthesis (6).

The preliminary studies reported above suggested that it is possible to control stress induced T1DM through systemic increase of insulin through NO synthesis by the neutralization of dermcidin.

In an effort to determine the role of dermcidin, if any, in the development of T1DM in humans, studies were conducted to determine the plasma levels of dermcidin in volunteers with the condition. Furthermore the pancreatic β cells are known to be non-functional in this condition. Therefore the feasibility of controlling hyperglycemia was attempted in the volunteers where the plasma levels of dermcidin were positively correlated with that of the plasma glucose levels, by the increase of hepatic insulin synthesis through the systemic stimulation of nitric oxide synthesis in human subjects with T1DM, similar to the alloxan induced insulin dependent diabetes mellitus in animal model, as reported before (7). The results of the investigation are presented herein.

## MATERIALS AND METHODS

### Ethical clearance

The protocol was approved by the Internal Review Board, Sinha Institute of Medical Science and Technology, Calcutta. This study also used normal white mice (*Mus musculus*) and adult New Zealand rabbits. Appropriate permission was obtained from the IRB.

As described in the Results section, adult volunteers with “confirmed” Type I diabetes mellitus, were asked to participate in this study. An appropriate permission was obtained from the Calcutta High Court of law (A.S.T 737 of 1999/ Appln # 2411/ Cal/ 97-F) after legal counseling was made available to the volunteers as described before (13).

### Chemicals

Goat anti-rabbit immunoglobulin G-alkaline phosphatase, HEPES (4-(2-hydroxyethyl)-1-piperazineethanesulfonic acid) were obtained from Sigma Chemical Co. Enzyme-linked immunosorbent assay (ELISA) Maxisorb plates were from Nunc, Roskilde, Denmark. Sodium nitroprusside was a product of Abraxis Pharm. All other chemicals were of analytical grade.

### Animals

White albino healthy mice (20-25 gm each), Swiss strain irrespective of gender as examined by a licensed veterinarian to determine that these animals were free from any diseases, were used for the study (14). These inbred animals were fed standard laboratory chow and sterilized water was given *ad libitum*. The animals were kept under 12h cycles of light and dark.

### Preparation of hepatocytes from the mice liver

Adult mice used in the study were killed by cervical dislocation and the liver was perfused by collagenase in a buffer consisting of 10 mM HEPES solution (pH 7.4) without the addition of glucose as described (9, 15).

### Preparation of the islets of Langerhans from the mice pancreas

The islets were prepared by incubating the pancreas in collagenase (9, 16). The islets were next separated by sedimentation (16), and suspended in Krebs bicarbonate buffer (pH 7.4), and used within 1 h of the preparation.

### Immunoblot analysis of insulin in the reaction mixture supernatant from the tissues of islets, hepatocytes, heart and kidney of mice

The presence or absence of insulin in the crude supernatant of islets of Langerhans, hepatocytes, heart and kidney from normal mice were identified by immunoblot technique in the presence and absence of glucose (17). The samples were subjected to SDS-polyacrylamide gel electrophoresis (PAGE) followed by staining with Coomassie brilliant blue (18). The separated protein bands were next transferred electrophoretically to a nitrocellulose membrane. Insulin was identified by using anti insulin antibody as described before (9).

### Amino acid sequence analysis of insulin produced in the liver

The immunopositive band that corresponded to the authentic insulin band in the PAGE was isolated from the gel and the amino acid sequence of the protein was carried out and identified by protein data bank matching as described before (6).

### Assay of NO

The determination of NO synthesis has been described before (19). The quantitation of NO was independently verified by chemiluminescence method (20).

### Determination of the glucose induced synthesis of insulin

The glucose induced stimulation of insulin synthesis was determined by incubating the islet preparation with 0.02 M of glucose (optimal amount for insulin synthesis) in Tyrod’s buffer (pH 7.4) for 30 min at 37°C. After incubation the nucleic acids which contained insulin mRNA was isolated (21) and translated as described (22). The amount of the synthesized insulin *in vitro* was determined by Enzyme Linked Immonosorbent Assay (ELISA) as described below.

### Selection of Type I Diabetes Mellitus Patients

The selected volunteers (n=45, M=25, F=20) between the ages of 30 and 45 years who had no history of systemic hypertension or suffered from cardiovascular or cerebrovascular condition participated in the study (Table 1). These volunteers were “confirmed” T1DM in that: (i) these patients had ≥170 mg/dL plasma glucose level after overnight (12 h) fasting, and 2 h impaired plasma glucose level following the guidelines of American Diabetes Association, and (ii) plasma insulin level were ≤10-15 µunits/dL, and, (iii) these subjects were suffering from the condition at least >1 year. The use of oral hypoglycemic agents had no effect on the hyperglycemia in these subjects. These volunteers for their personal and/or religious beliefs never received any insulin administration for the control of hyperglycemia. Diet control along with physical exercise were the main stays used by these subjects for the control of blood glucose level. Before they were asked to participate in the study, the plasma dermcidin level was determined by enzyme linked immunosorbent assay (ELISA) as described below. As it was essential for the intended study that these volunteers were free of liver diseases, as much as could be ascertained, volunteers with history of hepatitis, fatty liver, hepatomegaly, cirrhosis, neoplastic conditions were excluded from the study. All obese, alcoholics and heavy tobacco users were also excluded. All subjects were asked not to take any medication including paracetamol, which is known to damage liver cells, as well as acetyl salicylic acid at least for 3 weeks before they were asked to participate in the study. All the selected patients had Body Mass Index (BMI) ranging between 18.5 to 21.2 kg/m^2^. Patients maintained their hyperglycemia by the application of SNP “pad” as described in the paper in details, except that, the patients were recommended a brisk walking for 20min and to avoid any tobacco products during the entire study. Those who violated the request were automatically dropped from the study. The participants during the study were asked to determine blood sugar level using a glucometer twice a week.

**Table 1:** Clinical characterization of Type 1 diabetic patients selected for the study

Patients (M/F/Age in years)	BMI (kg/m^2^)	IFPG (mg/dL)	2h IPG (mg/dL)	Insulin (μunits/dL)	Dermcidin (nM)	Polyuria	Polydipsia	Family History

1. (M/34)	20.4	175	200	11.5	112.5	+	+	+
2. (M/32)	21.5	185	210	10.5	100.2	+	+	+
3. (M/23)	19.9	170	205	12.5	115.6	+	+	-
4. (F/23)	19.2	198	200	13.9	121.5	-	-	-
5. (F/24)	18.5	199	215	14.25	120.5	+	+	-
6. (M/26)	19.9	170	211	14	119.5	-	-	-
7. (F/28)	20.1	175	200	15	110	+	+	+
8. (M/31)	20.5	171	220	14.9	109.4	+	+	-
9. (F/32)	21.9	172	215	14.5	98.4	+	+	-
10. (F/29)	21.2	175	210	13	99.4	+	+	+
11. (M/28)	19.2	179	200	13.25	169	+	+	-
12. (F/28)	21	182	209	12.96	79	-	-	-
13. (F/30)	21.2	195	220	11.25	89.9	+	+	+
14. (M/25)	21	199	225	10.25	124.2	+	+	+
15. (M/26)	21.2	183	215	10	119.6	+	+	-
16. (F/24)	19.5	175	200	10.8	115.9	-	-	-
17. (M/28)	19.9	170	214	11	107.4	+	+	-
18. (M/29)	20.1	195	200	12.2	110.8	-	-	-
19. (F/31)	18.5	170	205	13.25	106	+	+	-
20. (M/30)	21	184	200	14.2	83.2	+	+	+
21. (M/33)	21	185	217	14	85.5	+	+	+
22. (F/34)	19.95	189	220	15.26	87.8	+	+	+
23. (M/35)	20.4	190	200	11	98.9	+	+	-
24. (M/32)	19.94	196	225	10.5	100.5	+	+	-
25. (M/35)	21.2	170	229	10.25	89.9	+	+	-
26. (F/24)	19.8	170	230	11.96	101.8	+	+	-
27. (M/36)	21.1	171	200	12.58	124.8	-	-	-
28. (F/34)	19.6	175	210	12.65	176	+	+	+
29. (F/32)	20.5	186	205	13	165	+	+	-
30. (M/30)	21	172	200	12	166	+	+	-
31. (F/29)	18.8	170	220	10.25	164	+	+	+
32. (F/28)	19.98	184	215	10.63	143	+	+	-
33. (M/25)	21.1	175	217	15	130	+	+	-
34. (M/32)	21	175	200	14.96	180	+	+	+
35. (F/26)	21.1	193	200	14	99.9	-	-	-
36. (F/34)	19.91	170	210	11.25	171.5	+	+	-
37. (M/27)	21	185	205	10	167.7	+	+	+
38. (M/29)	21	190	200	10	98.9	+	+	+
39. (F/30)	21.2	175	214	10.91	142.8	+	+	-
40. (F/32)	20.5	174	200	11.25	180	+	+	+
41. (M/33)	21	175	207	12	139.9	+	+	-
42. (M/35)	21	170	220	10	175	+	+	-
43. (F/29)	21.2	185	215	11.25	143.5	+	+	+
44. (M/24)	20.1	182	230	12.5	159.9	-	-	-
45. (M/32)	21	183	220	10.5	166	+	+	+

The characterization of the T1DM patients as presented in the table were carried out following the guidelines of the American Diabetes Association. M, male; F, female as given within the parentheses; IFPG, Impaired Fasting Plasma Glucose level; IPG, Impaired Plasma Glucose level; BMI, body mass index.

### The preparation of cell free plasma

Blood was drawn from the volunteers by venipuncture by using 19 gauge siliconized needles and collected in plastic vials in sodium citrate as anticoagulant (23), [9 vol blood: 1 vol of the anticoagulant (0.013 M final concentration)]. Cell free plasma (CFP) was prepared by centrifuging the blood sample at 30,000 g for 30 min at 0°C (23). The supernatant obtained was collected and used as CFP.

### Determination of the plasma dermcidin levels in T1DM subjects by ELISA

Dermcidin was quantitated by ELISA by using anti dermcidin antibody. Dermcidin used was prepared and purified by repeated electrophoresis as described before (6). The antibody against the purified dermcidin was raised in adult New Zealand rabbits after its emulsification with Freund’s adjuvant and used in ELISA assay for the quantitation of dermcidin by similar procedure as described under the ELISA for insulin. A standard curve for ELISA of dermcidin was constructed using elctrophoretically homogenous protein. As low as 0.029 pmol/ml and as high as 0.053 pmol/ml of dermcidin could be quantitated using the constructed standard curve. Whenever needed, the samples were appropriately diluted for the quantitation of the stress induced protein (6).

### Preparation of human dermcidin

Dermcidin used in this study was prepared from the plasma of the subjects suffering from acute ischemic heart disease by sequential purification by PAGE first in the presence of sodium dodecyl sulfate followed by reelectrophoresis in the absence of the detergent, followed by the elution of the protein band from the gel, and was used only after overnight dialysis as described before (6).

### Identification of dermcidin band in the cell free plasma from T1DM subjects

The cell free plasma from the T1DM subjects was electrophorosed on SDS-PAGE and the protein bands were stained by Coomassie brilliant blue (18). The molecular weight of the protein bands were determined by using Mr. marker proteins. The intensities of the protein bands were expressed in computer assisted pixel units.

### Preparation and the dermal application of sodium nitroprusside soaked cotton pad

Typically a small sterile absorbent cotton pad (25 mm × 10 mm) soaked in 1.6 M sodium nitroprusside dihydrate (SNP) solution in 0.9% NaCl containing 0.28 mmol of SNP “pad” was prepared (7) and the SNP “pad” was stuck to the hair free area of skin of the abdomen of the volunteers by using non toxic adhesive tape as described in details before (24). As needed, the SNP “pad” was replaced every 24 h. The application of the SNP “pad” did not produce hepatotoxic, teratogenic or changes in blood picture as described before (24).

### The rationale for using SNP “pad” for the systemic increase of plasma NO level

Nitric oxide released from the SNP in the skin cells has been reported to stimulate more NO synthesis through activation of insulin activated nitric oxide synthase (IANOS) in various cells including the formed elements of blood in the circulation (24). In other words, NO, the product of the IANOS, led to a rare “positive feedback” activation of the enzyme resulting in more systemic NO synthesis (24, 7).

### ELISA for insulin

Polyclonal antibody against pure insulin was raised in adult New Zealand rabbit by intradermal injection of insulin emulsified with Freund’s adjuvant. The antibody titre was determined by ouchterlony method (25).

The feasibility of the determination of insulin by ELISA was pretested by Immunoblot technique (17). The analytical precision of the assay for insulin was determined by “recovery” experiments that was found to be >90%.

### Determination of blood glucose level

The blood glucose level was determined by using a glucometer (Behringer).

### Statistical analysis

The results shown are mean±standard deviation (SD) of at least 5 experiments, each carried out in triplicate. The significance of the results was analyzed by Student’s t test. Significance (*p*<0.0001) was considered significant. The coefficient of correlation (r) was determined by Pearson test.

## RESULTS

### The effect of glucose on the synthesis of insulin in mice kidney, heart, liver and pancreatic cells

**Figure 1:**
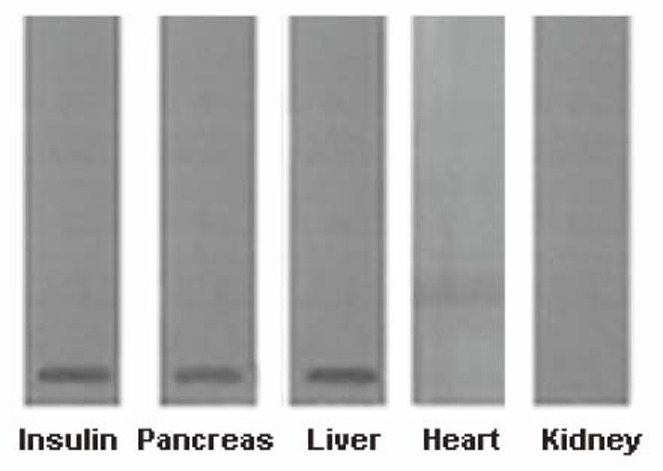
Immunoblot analyses of insulin band from different cell suspension from adult mice incubated with glucose using anti-insulin antibody. Islets of Langerhans and hepatocytes were prepared as described in the Materials and Methods; heart and kidney cells were prepared as described before (36) (25 mg/ml each) and incubated with 0.02 M glucose for 30 min at 37ºC. The supernatants were collected separately and subjected to SDS-PAGE. After the electrophoresis, immunoblot analysis was performed by using anti insulin antibody (16, 9). The figure shown is a typical representative of at least 5 different experiments using 5 different mice.

We have reported before that the treatment of epitroclearis muscles in the mice did not result in the synthesis of insulin as evidenced by immunoblot assay after appropriate PAGE analysis (9). Further experiments were carried out to determine the effect of glucose on the synthesis of insulin in heart and kidney tissues. As in the case of muscles, neither kidney nor heart tissues produced insulin that could be identified by immunoblot after PAGE (Fig. 1). Positive control experiment using both liver and pancreas were used both of which had shown the synthesis of insulin in the presence of glucose as reported before (9). These experiments were carried out to determine the effect of glucose on the synthesis of insulin like growth factor (IGF) with which insulin is known to cross react due to the presence of similar epitopes in their molecules. These experiments demonstrated that no IGF was produced by glucose.

The ELISA of insulin from the liver sample was verified by bioassay as described before (9). The synthesis of insulin when treated with glucose was also verified by amino acid sequence analysis from the liver of the isolated protein which conclusively proved that the protein was insulin upon amino acid sequence matching (Protein Databank).

Finally, the mRNA produced from the liver cells that was used to make cDNA with base pairs 182 and 188 corresponded to the two non-allelic proinsulin I and II genes respectively (9). No IGF cDNA was present in the proinsulin cDNA preparation. These results demonstrated that the protein molecules produced in the liver was indeed insulin and was not IGF that could be erroneously thought to be insulin.

### Glucose induced synthesis of NO in the hepatocytes and in the islets of Langerhans

Glucose which is well known for its ability to synthesize and secrete insulin in the islets of Langerhans (1) and in the hepatocytes (9), was found to be a potent inducer of nitric oxide synthase (NOS) in both the hepatocytes and the islets of Langerhans. It was found that the treatment of hepatocytes or the islets with 0.1 M glucose increases the synthesis of NO from 0.244 ± 0.021 nM to 3.132 ± 0.212 nM after 30 min of incubation as described in the Materials and Methods indicating the stimulation of NO synthesis in these cells. And, as such, glucose itself, like aspirin, was capable of stimulating the synthesis of NO. The glucose induced NO synthesis would also help in the conversion of proinsulin to insulin by the formation of plasmin in the expression of proinsulin genes as described above. It was also found that the inhibition of NO synthesis resulted in the inhibition of the secretion of insulin as reported before (7) suggesting that glucose also had an important role in the secretion of insulin through the synthesis of NO beyond the actual synthesis of the hormone (1).

### Plasma levels of dermcidin in T1DM subjects

**Figure 2:**
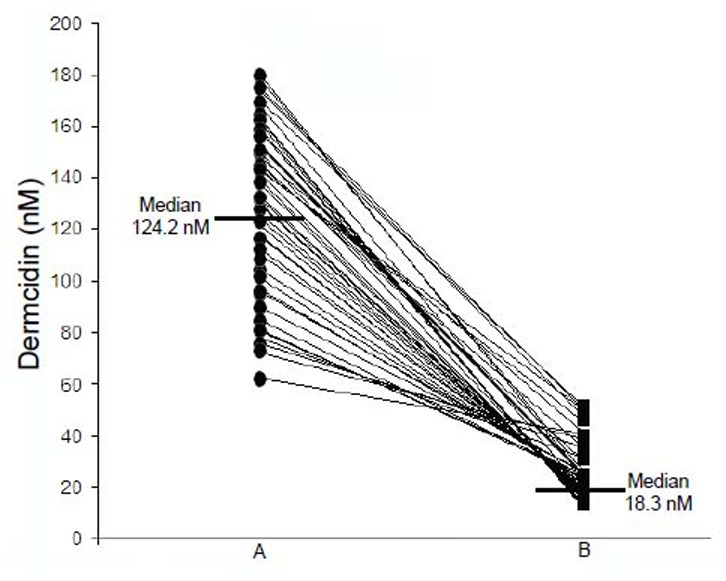
The plasma dermcidin levels in T1DM subjects before and after the application of the SNP “pad” for 30 days. Cell free plasma was prepared from the blood samples obtained from T1DM patients (n=45, F=20, M=25). Ten of these volunteers dropped out of the study. The plasma dermcidin levels were determined in 27 of the volunteers (F=15, M=12) by ELISA as described before. A represents dermcidin level in Type I diabetes patients before the application of SNP “pad”; B represents dermcidin level the same patients after the application of SNP “pad”.

**Figure 3:**
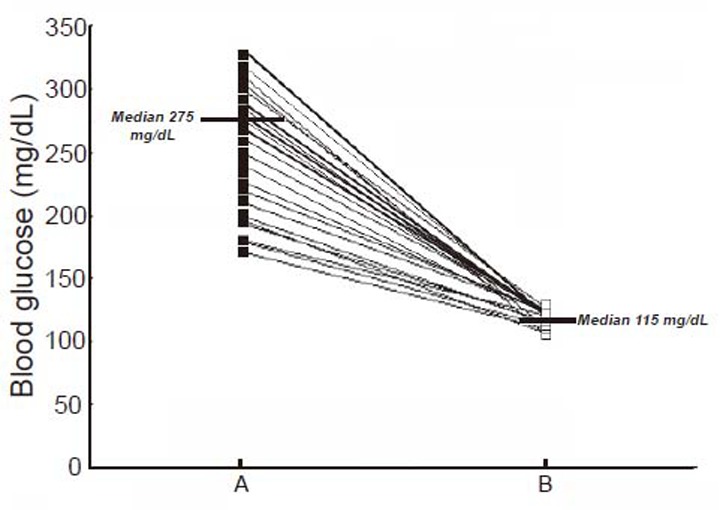
The control of hyperglycemia in T1DM subjects by the dermal application of SNP “pad” for 30 days. The selected volunteers with T1DM who had increased dermcidin levels as described under Fig. 2 were asked to use dermal SNP soaked pad as described in the Materials and Methods. The SNP “pad” was changed every 24 h. The blood sugar level were monitored every 24 h. After 30 days of continuous application of SNP, blood sugar levels (Panel B) were compared with the blood glucose level before the application of SNP (Panel A).

To determine whether the stress induced elevation of the plasma levels of dermcidin in T1DM could have any role in the development of the condition, the plasma levels of dermcidin were determined in the blood of the volunteers with TIDM and compared with those in age and sex matched normal volunteers (Fig. 2, Panel A). It was found that the plasma dermcidin level in majority (65%; *p*<0.0001) of the T1DM subjects had higher levels of the stress induced protein in the plasma when compared with that in normal controls. It was also found that the plasma dermcidin (Fig. 2, Panel A) and the blood sugar levels in T1DM were highly and positively correlated (Fig. 3, Panel A) (coefficient of correlation “r”= +0.9899; (Pearson) *P*<0.0001).

We have reported before that the presence of dermcidin in the reaction mixture containing either hepatic cells or the islets of Langerhans from the adult mice resulted in the inhibition of glucose induced insulin synthesis due to the inhibition of glucose uptake in the presence of the stress induced protein in the reaction mixture (6, 7). The stimulation of NO synthesis in these tissues was found to result in the restoration of glucose uptake which in consequence was found to restore the synthesis of insulin leading to the control of hyperglycemia in alloxan treated diabetic mice where the synthesis of insulin in the pancreatic cells were completely abolished in the control experiments (7). Similar results were obtained by the direct addition of NO solution (2.5 μM) in 0.9% NaCl which restored the synthesis of the hormone due to the restoration of glucose uptake with concomitant expression of both proinsulin I and II genes in the hepatic cells (7).

Based on these results efforts were made to control hyperglycemia in humans with TIDM by stimulating systemic NO synthesis by the application of SNP “pad” on the skin of the participating subjects as in the case of animal experiments (7).

### The effects of the dermal application of SNP “pad” in TIDM subjects on the plasma NO and dermcidin levels and on the control of hyperglycemia related to the increased plasma insulin level

**Figure 4:**
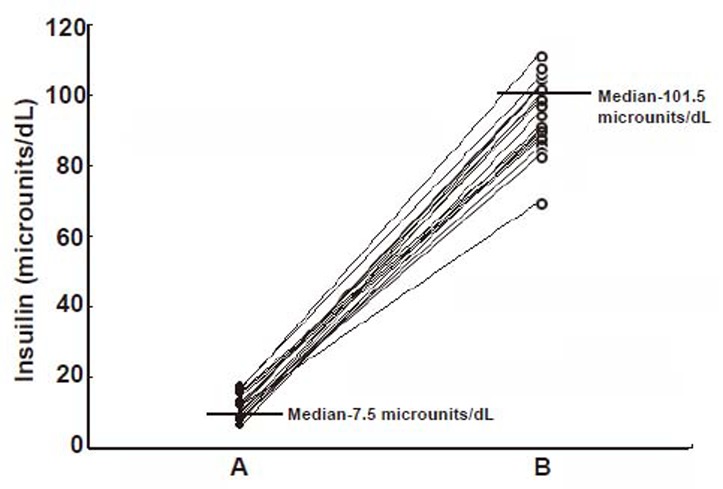
The plasma insulin levels in T1DM subjects before and after the application of the SNP “pad” for 30 days. The details of the selected volunteers with T1DM have been described under Table 1. The blood glucose levels of these volunteers have been described under Fig. 3. In these same patients, as described in Fig. 3, the plasma insulin levels were determined by ELISA of the hormone before (Panel A) and after (Panel B) using SNP as described in the Materials and Methods.

The determination of the plasma NO and dermcidin levels at different time after the application of the SNP “pad” in TIDM subjects demonstrated that the increase of the plasma NO levels resulted in the decrease of the plasma dermcidin level, and at 24 h after the application of the dermal SNP “pad”, when the plasma NO reached its maximal level, the plasma dermcidin level was found to be decreased to normal when compared with controls. The plasma dermcidin level which was 126.6 ± 10.5 nM before the application of SNP “pad” decreased to normal plasma level of 11.89 ± 2.5 nM after 24 h with concomitant increase of NO from 0.43 ± 0.19 nM at 0 h to 4.1 ± 1.56 nM after 24 h of the application of the dermal SNP “pad” (*p*<0.0001; n=45). The application of SNP “pad” was continued for 30 days to achieve the systemic equilibrium. It was found that the dermcidin level which was 124.2 nM (median, ranging from 180.2 to 62 nM) before the application of the SNP “pad” decreased to 18.3 nM (median, ranging from 50.3 to 12.2 nM) after the application of the “pad” (Fig. 2, Panel A and B). Also, compared to day 1, the NO level which was 0.39 ± 0.12 nM increased to normal ranges of 4.5 ± 1.45 nM after the dermal use of the SNP “pad” for 30 days. In these same subjects when both the blood sugar and insulin levels were determined, it was found that the hyperglycemia was controlled [i.e., the blood glucose level was found to be reduced from 275 mg/dL (Median) to 115 mg/dL (Median)] (Fig. 3) with the concomitant increase of plasma insulin levels [from 7.5 µunits/dL (Median) to 101.5 µunits/dL (Median)] (Fig. 4). It was also found that among these participants with T1DM (n=45), 4 of these volunteers did not respond to the application of the dermal SNP “pad”. In these “non responders” subjects, the application of SNP neither controlled hyperglycemia nor resulted in the increase of plasma insulin level. In contrast, it was also noted that the application of SNP “pad” in 5 “responder” volunteers were unexpectedly “cured” of their T1DM (*p*<0.0001), in that, they did not need daily application of the SNP “pad” for the control of hyperglycemia after the application of the SNP “pad” for consecutive 30 days. In contrast, majority of the T1DM subjects (≈36 persons) in our study needed daily application of SNP “pad” for the control of hyperglycemia (*p*<0.0001).

**Figure 5:**
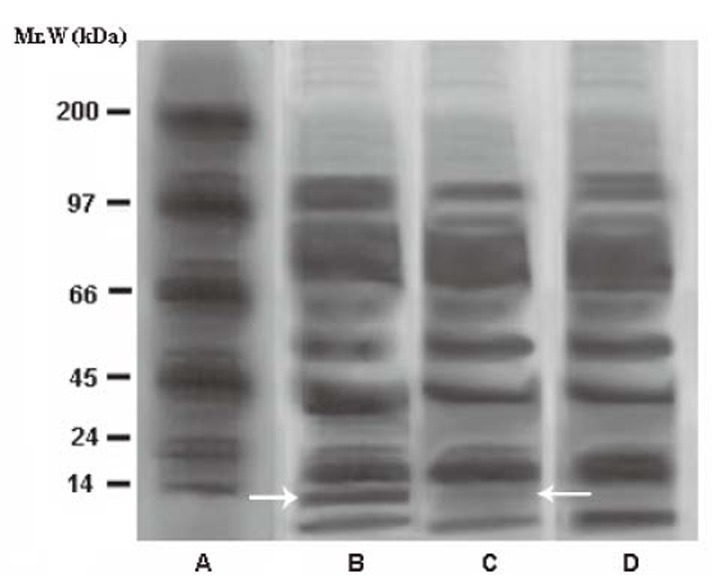
The effect of application of SNP “pad” on the intensity of the dermcidin band on SDS-PAGE of the plasma from T1DM subjects for 24h. Panel A: shows molecular marker protein bands; Panel B represents the cell free plasma from T1DM subjects before the application of the dermal SNP “pad”; Panel C indicates the cell free plasma from the same T1DM subjects after the application of SNP “pad”; Panel D: shows the cell free plasma from normal volunteers; The white arrows indicate the dermcidin bands in SDS-PAGE. The white arrow in Panel B indicates dermcidin band before the application of the SNP “pad” and the white arrow in Panel C represents dermcidin band after the application of the SNP “pad”. The intensity of the dermcidin bands before and after the application of SNP is expressed in pixel units. The figure is a typical representative of at least 6 similar experiments using plasma from 6 different subjects in each group.

When the SDS-PAGE of the plasma from T1DM patients was carried out before and after the application of SNP “pad” for 24 h, it was found that the intensity of the dermcidin band (Mr. 11kDa) was markedly reduced after the application of the “pad” compared to that observed before the application of the compound to the abdominal skin of the volunteers as described above (Fig. 5). The computer assisted determination of the intensities in pixel units of the protein bands corresponding to dermcidin in the SDS polyacrylamide gels (white arrow in Fig. 5, Panel B) demonstrated that the intensity of dermcidin band which was 115 pixels before the application of SNP “pad” in the subjects with T1DM was found to be reduced to 47.0 pixels after the application of the SNP “pad” in the same subjects after 24 h (Fig. 5, Panel C). These results indicated that the reduction of the plasma dermcidin level in T1DM subjects was related to the application of SNP “pad”. In a separate experiment, the plasma NO level was also found to increase by >6 folds as described above.

## DISCUSSION

These results suggested that the hepatocytes as in the case of pancreatic β cells in adult mammals were also capable of synthesizing and secreting insulin, when stimulated by glucose. However unlike the pancreatic cells in T1DM, the synthesis of insulin could be normalized by the hepatic synthesis of the hormone through the increased systemic NO level in both the alloxan induced diabetes mellitus in animal model (7) where the pancreatic β cells are reported to be completely destroyed or in the case of humans as presented above with T1DM where the β cells are reported to be destroyed (26). The above results suggested that although the pancreatic β cells in T1DM were destroyed, the glucose induced synthesis of insulin by the liver cells in human remained largely unaffected in that, the hyperglycemia could be corrected in this condition by the increase of hepatic insulin synthesis due to the stimulation of NO synthesis even in the absence of external insulin administration.

The role of NO in the conversion of proinsulin to insulin by the systemic increase of plasmin has been reported before (27-29). In that, NO plays a critically important role in the conversion of proinsulin to insulin in the liver cells. In the above context, it has been reported before that for the conversion of proinsulin genes product, i.e., proinsulin, to bioactive insulin, several convertases including PC1/3 and PC2 followed by the action of carboxypeptidase are needed (30). As these enzymes are not present in the liver, in consequence, proinsulin produced in the liver would remain in the proinsulin form and not converted into the bioactive insulin (30). In contrast to these reports, it has also been reported that proinsulin was converted to insulin in the presence of plasmin (27, 29). Similar results were also reported elsewhere (28) that described, “Various trypsin like serine proteases such as kallikreins and plasmin or plasminogen activator have been suggested to be involved in the cleavage of the hormone precursors”. Also it was mentioned that, “Cleavage of proinsulin by plasmin is shown to result in equal amounts of insulin and a form of insulin with a residual Arg residue before the Gly at the N-terminus of the A-chain”. As described above our results confirmed the above reports on the conversion of proinsulin to insulin by the formation of plasmin to plasminogen due to the activation of the proenzyme to active serine enzyme by NO by aspirin (7) Furthermore, the production of insulin by the hepatic cells was confirmed both by bioassay and by the amino acid sequence analysis of the isolated hormone and liver was no exception in that proinsulin was also converted to insulin in these cells. We have also reported that activation of plasminogen to plasmin by NO in the absence of any cells or cofactor, and independent of the classical Hageman factor dependent formation of plasmin in the intrinsic pathway of blood coagulation (31).

The role of autoimmunity in the destruction of the pancreatic β cells leading to T1DM has been repeatedly claimed before (11, 26) although stresses are known to cause T1DM (32) whether the autoimmunity was also involved in the stress induced pathogenesis of the condition is not known. Our results however suggested that the systemic production of dermcidin, a stress induced protein produced both in the brain cells and in the endothelial cells inhibited insulin synthesis both in the islets of Langerhans and in the hepatocytes in adult mice (7). As such, it is possible that dermcidin could be one of the risk factors for the development of T1DM in humans specifically in type 1B diabetes mellitus (T1BDM) which lacks immunological marker (33). However as reported above, the number of dermcidin associated occurrence of T1DM in volunteers who participated in our study were the majority (>65%) among these volunteers. Whether the occurrence of large number (>65%) of patients in our study who had increased dermcidin leading to T1DM was related to the ethnic background representing T1BDM (33) or was it more universal in nature is not known. These results nevertheless might also suggest that the occurrence of autoimmunity was not the major cause of T1DM across all ethnic classes around the world. In this context it should also be mentioned that the dermcidin induced inhibition of insulin synthesis was not due to the inhibition of protein synthesis (i.e. insulin synthesis) related to the expression of insulin genes, but was related to the inhibition of glucose transport which is known to stimulate both the synthesis and the secretion of the hormone (34). In this sense dermcidin was found to be equally effective in the inhibition of insulin synthesis through the inhibition of glucose uptake both in the pancreatic islets and in the liver cells (6, 7). It was found that the inhibition of glucose uptake was mediated through the decrease of NO synthesis. In that, the inhibition of NO synthesis resulted in the inhibition of glucose uptake. However, unlike in the case of the autoimmunity induced destruction of the pancreatic β cells as in the case of the T1DM, the dermcidin induced inhibition of insulin synthesis could be neutralized through the stimulation of systemic NO synthesis (7) and, as such, the development of the condition induced by dermcidin not necessarily destroyed the synthetic ability of the hepatocytes to produce insulin. Our results also demonstrated, that the pathological consequences that led to T1DM in human, although destroyed the pancreatic β cells, might spare the synthetic ability of the hepatic cells to produce insulin in the presence of glucose. In the above context, it should be mentioned that dermcidin was a competitive inhibitor of all known forms of nitric oxide synthases (35) where *l*-arginine is the only known substrate, and the systemic inhibition of nitric oxide synthesis could be involved in the inhibition of insulin synthesis due to the inhibition of glucose transport by dermcidin. In the context of the importance of NO in hepatic insulin synthesis (7), the presence of glucose in the reaction mixture containing either the islets of Langerhans or the hepatocytes preparation was found to result in the synthesis of NO suggesting that glucose itself was an activator of nitric oxide synthase (NOS), although markedly weaker than acetyl salicylic acid in these cells, and the sugar itself might facilitate the transport of glucose through NO synthesis. It may also be mentioned here that insulin itself, a potent activator of NOS (36) might actually have a critical role in the prevention of the dermcidin induced T1DM through the neutralization of the stress protein induced inhibition of glucose transport due to stimulated NO production. As reported before, the addition of NO solution itself to the reaction mixture was able to neutralize the inhibitory effect of dermcidin in glucose uptake (6).

The affect of acetyl salicylic acid on the stimulation of insulin synthesis in the presence of glucose in the liver through the neutralization of the inhibitory effect of dermcidin (7) bought about an important corollary in the control of T1DM in humans by the compound. Due to various reasons, we could not carry the study to determine the use of aspirin in the control of T1DM in humans. However, in an independent study involving patients suffering from Acute Myocardial Infarction (AMI) where hyperglycemia is reported to produce worst prognostic outcome of the patients, the use of aspirin as a part of therapeutic regime for AMI for the control of hyperglycemia through insulin synthesis was determined (6). It was found that the AMI subjects (n=29) who had no history of diabetes mellitus were overtly hyperglycemic (>130 mg/dL ) with concomitant reduction of insulin level (19 to 0 µunits /dl) that accompanied the increase of plasma dermcidin level from normal (basal) 9 nM to 112 nM (*p*<0.0001) . Administration of 350 mg/kg body weight was found to normalize the blood sugar level to 100-120 mg/dL with increase of plasma insulin level (143 μunits/dL) with simultaneous normalization of dermcidin level (9.65 nM) compared to the control patients who did not receive any aspirin. It should be noted here that the effect of aspirin in these cases was not due to the inhibition of cyclooxygenase but due to the systemic increase of plasma NO level (23). These results suggested that the oral administration of aspirin might be helpful for the control of hyperglycemia in humans in T1DM in humans.

As described above, there were “non responders” among the volunteers with T1DM where the application of SNP had little effect either on the hyperglycemia or on the plasma insulin level. The mechanism of the failure of SNP is not known currently. However, the “functional” hepatocytes would be expected to play a critical role in the hepatic insulin synthesis, particularly when the pancreatic cells were destroyed for the control of hyperglycemia. It might be also possible that the failure of SNP was related to the presence of undiagnosed hepatic diseases in these volunteers. This is particularly relevant due to the fact that innumerable medications, infections, and viruses are known to damage liver cells (37-39). On the other hand our results suggested that T1DM in human was not necessarily always an irreversible disease particularly where the increased dermcidin level might have a critical role in the genesis of the disease. The systemic increase of NO level might even “cure” the condition in some cases where the inhibition of NO synthesis was related to the systemic increase of dermcidin level, and the increase of systemic NO level in these cases resulted in the normalization of plasma dermcidin level in humans (6) that led to increased insulin synthesis in the liver cells as described above. The “curing” of dermcidin induced T1DM might be related to transient impairment of systemic NOS activity. The occurrence of impaired NOS activity has been reported before in different conditions including cancer (24) and hypertension (35).

As discussed above, various risk factors are involved in the development of T1DM. Our study focused only on the role of a stress induced protein *viz*. dermcidin and the neutralization of its inhibitory effect on insulin synthesis by systemic increase of NO level. It has been reported before that unlike in type 1A diabetes mellitus (T1ADM), which usually resulted from the autoimmune β cell destruction, type 1B diabetes mellitus (T1BDM) resulted from unknown mechanism where immunological markers were not present (33). It was also reported that many of the T1BDM patients were of Asian origin. And, as such, stress induced appearance of dermcidin might be involved in the pathogenesis of T1BDM in the majority (>65%) of the patients who participated in our study.

From these results it can be concluded that the use of dermal application of SNP “pad”, that increased insulin synthesis in the liver even when the pancreatic β cells are non-functioning, could possibly be an alternative modality for the control of hyperglycemia in T1DM without using external insulin, and, therefore could be therapeutically useful in this condition.
